# Chanarin-Dorfman Syndrome with Multi-System Involvement in Two Siblings

**DOI:** 10.4274/tjh.93798

**Published:** 2013-03-05

**Authors:** Seçil Arslansoyu Çamlar, Pınar Gençpınar, Balahan Makay, Ayşe Yüzbaşıoğlu, Nur Arslan, Serap Emre Dökmeci, Özden Anal, Galip Köse

**Affiliations:** 1 Dokuz Eylul University School of Medicine, Department of Pediatrics, İzmir, Turkey; 2 Hacettepe University School of Medicine, Department of Medical Biology, Ankara, Turkey

**Keywords:** Ichthyosis, child, Chanarin-Dorfman syndrome

## Abstract

Chanarin-Dorfman syndrome (CDS) is a very rare autosomal recessive inherited neutral lipid metabolism disorder associated with congenital ichthyosis and multi-system involvement. Observation of lipid vacuoles in neutrophils (Jordan’s anomaly) in peripheral blood smears in patients with ichthyosiform erythroderma is diagnostic. Herein we present 2 siblings with CDS that were referred to Dokuz Eylul University School of Medicine Department of Pediatrics due to ichthyosis. They had hepatomegaly, cataract, growth retardation, and sensorineural hearing loss. Some lipid vacuoles in neutrophils were noted in peripheral blood smear evaluation. Genetic analysis showed homozygous N209X mutation in both patients. They were put on a low-fat high-carbohydrate diet supplemented with medium-chain fatty acids. During 6 months of follow-up, no improvement was observed in both patients. In conclusion, although CDS is a rare lipid storage disease, it should always be a consideration in patients with congenital ichthyosis, especially those with extracutaneous symptoms or signs. The diagnosis of CDS is made based on a very simple test-peripheral blood smear.

**Conflict of interest:**None declared.

## Abstract (tr)

Chanarin-Dorfman Sendromu (CDS) konjenital iktiyozis ve çoklu organ tutulumunu ile giden, otosomal resesif geçişli, nadir görülen bir nötral lipid metabolizması bozukluğudur. İktiyoziform döküntüleri olan hastalarda periferik kan yaymasında nötrofillerdeki lipid vakuollerinin (Jordan anomalisi) gösterilmesi ile tanı konur. Bu yazıda iktiyozis nedeniyle bölümümüze yönlendirilen ve hepatomegali, katarakt, büyüme geriliği, sensörinöral işitme kaybı eşlik eden, periferik kan yaymasında nötrofillerde lipid vakuolleri saptanarak CDS tanısı alan iki kardeş sunuldu. Yapılan genetik çalışmada her iki hastada da homozigot N209X mutasyonu saptandı. Hastalara tedavi amacıyla orta zincirli yağ asitleri ile zenginleştirilmiş ve yağ oranı düşük, karbohidrat içeriği yüksek olan bir diyet programı başlandı. Sonuç olarak, CDS oldukça nadir görülen bir lipid depo hastalığı olmasına rağmen, konjenital iktiyozlu özelikle de cilt dışı organlarda da tutulum bulguları olan her hastada akılda tutulmalıdır. Hastalığın tanısı periferik kan yayması gibi çok kolay bir testle konulabilir.

## INTRODUCTION

Chanarin-Dorfman syndrome (CDS, OMIM: 275630) is a rare autosomal recessive inherited neutral lipid metabolism disorder associated with ichthyosis and multi-system involvement [1,2]. It is characterized by congenital ichthyosiform erythroderma, vacuoles in leukocytes (Jordan’s anomaly), and variable involvement of the liver, muscles, and central nervous system [2]. Systemic involvement may manifest as hepatosplenomegaly, bilateral cataracts, growth retardation, myopathy, ataxia, bilateral sensorineural hearing loss, and horizontal nystagmus [2]. Observation of lipid vacuoles in neutrophils in peripheral blood smears in patients with ichthyosiform erythroderma is diagnostic [2,3]. Mutations of the ABHD5 gene have been identified as the cause of CDS. The human ABHD5 (CGI-58) gene, which is located on chromosome 3p21, encodes a protein of the esterase/lipase/thioesterase subfamily. This gene product co-activates adipose triglyceride lipase (ATGL), which controls hydrolyzation of triacylglycerol. Herein we present 2 siblings with generalized ichthyosiform erythroderma that were diagnosed as CDS based on observation of lipid vacuoles in neutrophils in peripheral blood smear evaluation. Additional mutation analysis confirmed the diagnosis. 

## CASE REPORT

A 9-year-old girl and her 11-year-old brother were referred to our hospital for evaluation of ichthyosiform eruptions that have occurred since birth. They both suffered from non-bullous ichthyosiform lesions, with exacerbation in winter and after bathing. The patients were being treated with emollients, but skin scaling was persistant. The boy also had myopia and hearing loss, which began 1 year earlier. Both patients were born full-term via normal vaginal delivery to second-degree consanguineous parents. No other family member had similar eruptions. 

**Figure 1:**
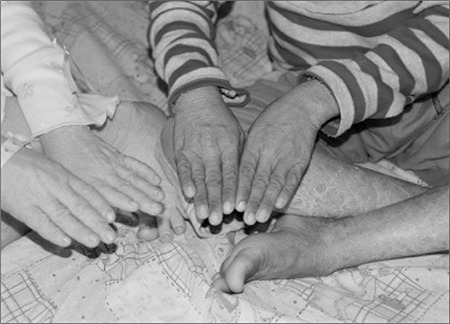
Mild erythema and fine desquamation of the skin especially on the extensor surfaces of the extremities.

**Figure 2:**
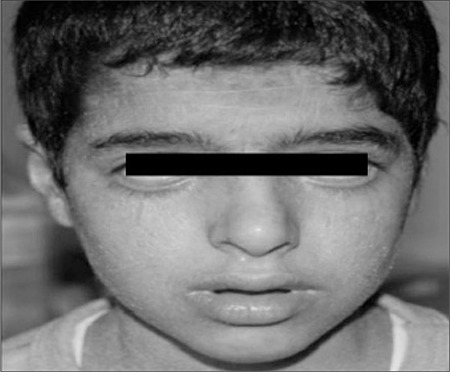
Bilateral ectropion due to dry eye obtained additionaly in the male patient.

On physical examination both their weights and heights were below the 3^rd^ percentile (Table). They had mild erythema and fine desquamation of the skin especially on extensor surfaces of the extremities (Figure 1). They had firm, non-tender hepatomegaly palpable 2 cm below the right costal margin. They did not have splenomegaly. No evidence of muscle weakness was noted and neurological examination findings were normal. Cardiovascular and respiratory system examinations were within normal limits. In both patients astigmatism and cataract, and bilateral moderate sensorineural hearing loss were noted on ophthalmological and otorhinolaryngological examination, respectively. The boy also had bilateral ectropion due to dry eye (Figure 2). Unfortunately, bone age of the patients was not estimated. 

**Figure 3:**
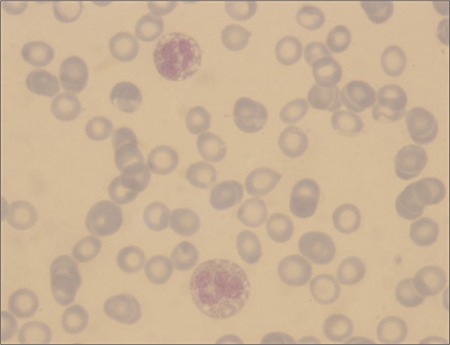
The peripheral blood smear revealed lipid vacuoles in granulocytes.

Complete blood count, urinalysis, serum electrolytes, protein, and albumin were within normal limits. Peripheral blood smear in both patients showed lipid vacuoles in granulocytes (Figure 3). Serum alanine aminotransferase and creatine phosphokinase in both patients were elevated. Serum lipid profiles were within normal limits and serum biotinidase activity was normal. The patients’ laboratory results are summarized in the Table. Abdominal ultrasonography showed an enlarged liver with fatty infiltration in both patients. Stool pH was normal and stool fat excretion was negative in both patients. Electromyography and muscle biopsy was scheduled, but the parents did not consent to these evaluations. Both patients had mild mental retardation based on WISC-R test. The parents’ peripheral smears did not show vacuolization in eosinophils. 

After obtaining informed consent from the parents, the patients’ DNA was isolated from peripheral leukocytes. Complete DNA sequencing of the 7 exons and the intron/exon boundaries of the ABHD5 gene (CGI-58 gene) was performed. PCR was performed as previously described [4]. Cycle sequencing was performed using an ABI PRISM Big Dye Terminator Cycle Sequencing Kit, according to the manufacturer’s instructions, and sequences were analyzed using an ABI PRISM 3130 DNA analyzer. Genetic analysis showed that both patients had homozygous N209X mutation. 

The patients were put on a special diet and multivitamin program to treat their malnutrition and other symptoms. This diet included 15% protein, 65%-70% carbohydrates, and 20% fat (18% middle chain triglycerides and 2% long chain triglycerides). Ursodeoxycholic acid and vitamin E were administered for their hepatic cytoprotective and antioxidant effects, respectively. Local application of emollients containing urea was continued for the treatment of ichthyosis. In six months follow-up, they had no clinical improvement. 

## DISCUSSION

Two siblings were diagnosed as CDS based on physical examination, peripheral blood smear findings, and mutation analysis. CDS is a rare autosomal recessive inherited lipid storage disease associated with congenital ichthyotic erythroderma, and is characterized by neutral lipid accumulation in different organs, such as skin, muscle, the liver, central nervous system, and granulocytes [2,3,5]. To the best of our knowledge nearly 40 patients with this rare disorder have been described to date. 

Accumulation of lipid droplets in various tissues is related to abnormal catabolism of triacylglycerols. Neutral lipid, in the form of triacylglycerol, accumulates in leukocytes, fibroblasts, the liver, muscle cells, and intestinal mucosa as non-membrane-enclosed cytoplasmic droplets [6]. These droplets can be observed in peripheral smears as vacuoles in granulocytes and monocytes, which is known as Jordan’s anomaly [6,7]. CGI-58 protein is located on the surface of cytoplasmic lipid droplets. It improves the catabolism of stored fat in adipose tissues by working in conjunction with perilipin and adipose triglyceride lipase (ATGL) [1,8,9,10]. When perilipin is activated by CGI-58, hormone-sensitive lipase activity is stimulated, leading to lipolysis [9]. Thus, mutation in the CGI-58 gene (ABHD5 gene) interferes with lipolysis and leads to accumulation of lipid droplets and, consequently, CDS.

Different degrees of liver involvement in CDS have been reported. The liver is clinically affected in 64% of CDS cases; however, steatohepatitis in liver biopsy specimens and elevated liver enzymes are reported all of the (100%) CDS patients with or without hepatomegaly [6,11]. Liver biopsy could not be performed in the presented patients, but transaminase levels were high, and hepatomegaly and hepatosteatosis were observed on physical examination and ultrasonographically, respectively. 

Homozygous N209X mutation was noted in both patients. Emre et al. [12] reported that N209X mutation was the most common mutation (58.3%) in Turkish CDS patients. To date, 4 Turkish patients with homozygous N209X mutation and 1 with heterozygous N209X mutation have been reported [4,12]. This mutation is predicted to have a negative effect on both CGI-58 structure and function. The N209X nonsense mutation causes premature termination of translation, and results in a truncated 140-amino acid peptide instead of the normal protein. This mutation further illustrates the functional importance of CGI-58 in lipid metabolism and epidermal differentiation. Determination of the correlation between the phenotypic and genotypic characteristics remains difficult, as most ABHD5 mutations are novel and unique.

Management of patients with CDS is difficult. Emollients are useful for local treatment [13]. Kakourou et al. [14] prescribed a low-fat diet to their CDS patient and observed improvement in skin and liver manifestations during 1 year of the therapy. The presented patients were prescribed a diet of 15% protein, 65%-70% carbohydrates, and 20% fat (18% middle chain triglycerides and 2% long chain triglycerides). During 6 months of follow-up no improvements were observed in either patient. In conclusion, although CDS is a rare lipid storage disease, it should be a consideration in every patient with congenital ichthyosis, especially those with extracutaneous signs, as a simple peripheral smear can establish the diagnosis.

**Conflict of Interest Statement**

The authors have no conflicts of interest relevant to the materials presented in this manuscript.

## Figures and Tables

**Table 1:**
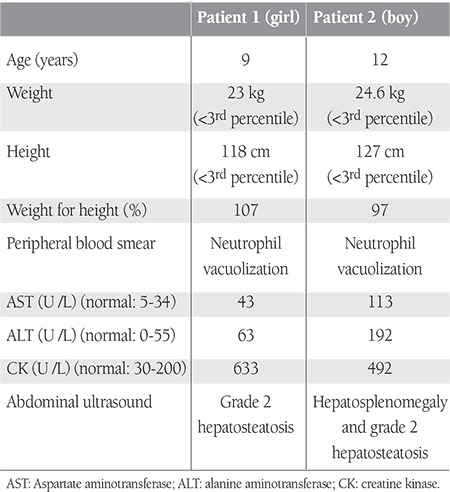
Patient clinical and laboratory data.
